# Gynecologic perivascular epithelioid cell tumors (PEComas): a review of recent evidence

**DOI:** 10.1007/s00404-024-07510-5

**Published:** 2024-04-25

**Authors:** Gabriel Levin, Mariana Pilon Capella, Raanan Meyer, Yoav Brezinov, Walter H Gotlieb

**Affiliations:** 1 Department of Gynecologic Oncology, Jewish General Hospital, McGill University, Montreal, QC Canada; 2 Department of Oncology, Brazilian Institute for Cancer Control, São Paulo, SP Brazil; 3 Division of Minimally Invasive Gynecologic Surgery, Department of Obstetrics and Gynecology, Cedar Sinai Medical Center, Los Angeles, CA, USA; 4 Experimental Surgery, McGill University, Montreal, Canada

**Keywords:** PEComa, mTOR, Gynecologic oncology, Surgery, Rare disease

## Abstract

Gynecologic perivascular epithelioid cell (PEC) tumors, or 'PEComas,' represent a rare and intriguing subset of tumors within the female reproductive tract. This systematic literature review aims to provide an updated understanding of gynecologic PEComas based on available literature and data. Although PEComa is rare, there are varied tumor-site presentations across gynecologic organs, with uterine PEComas being the most prevalent. There is scarce high-quality literature regarding gynecologic PEComa, and studies on malignant PEComa underscore the challenges in diagnosis. Among the diverse mutations, mTOR alterations are the most prominent. Survival analysis reveals a high rate of local recurrence and metastatic disease, which commonly affects the lungs. Treatment strategies are limited, however mTOR inhibitors have pivotal role when indicated and chemotherapy may also be used. with some cases demonstrating promising responses. The paucity of data underscores the need for multicentric studies, an international registry for PEComas, and standardized reporting in case series to enhance clinical and pathological data.

## Introduction

Gynecologic perivascular epithelioid cell (PEC) tumor–‘PEComa’ was first described in 1996 [1] in a 57 years old female with an abnormal bleeding and a polypoid lesion protruding in the uterine cavity. PEComas are a rare and intriguing group of tumors that can arise within the female reproductive system. They are characterized by their unique histological features, composed of perivascular epithelioid cells that exhibit both smooth muscle and melanocytic differentiation [2]. These tumors can occur in various gynecologic organs, including the uterus, ovaries, and vulva [3]. While most gynecologic PEComas are benign, some have the potential to behave in a malignant or aggressive manner [4]. Management typically involves surgical resection, and the prognosis depends on factors such as tumor size, location, and histological characteristics. However, no clear guidelines exist [5]. Given their rarity, the understanding of gynecologic PEComas is still evolving, and ongoing research is essential to better comprehend their pathogenesis and guide treatment strategies. Most literature regarding gynecologic PEComas is composed of case reports and small case series, and PEComa can coexist with other pathological entities, such as leiomyoma and fumarate hydratase-deficient atypical leiomyoma [6]. In light of the rarity of the diagnosis, coupled with abundance of case-reports and few available original studies, there is a gap of concise relevant synthesis of data. We aim to systematically review the published literature regarding PEComa, and to abstract the available data from studies, excluding reviews and case reports.

## Materials and methods

A search was performed in PubMed database using a combination of the medical subject heading terms (MeSH): “PEComa” AND "gynecologic neoplasm”. We have searched the database since inception to 31st Oct. 2023.

**Fig.1:**
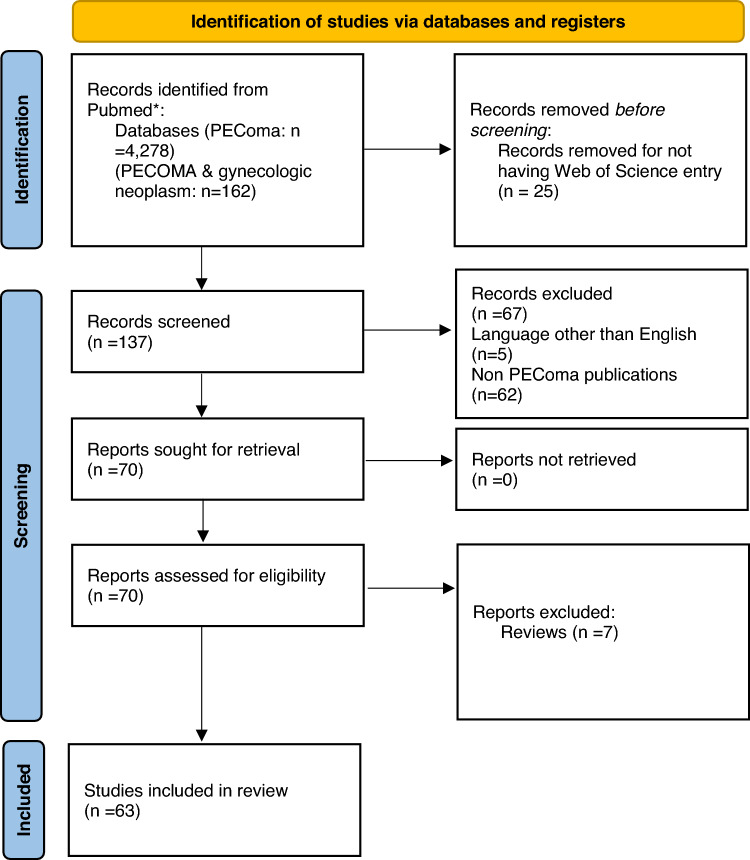
PRISMA 2020 flow diagram on the included publcations

The PRISMA guideline was used in the preparation of this review (Fig. 1) [7]. All the Mesh search results Pubmed-indexed publications were entered into a database. These publications were retrieved for their records in the Web of Science database by search field Pubmed ID in the Web of Science platform. The results from this search were then manually reviewed. We manually reviewed each title and abstract for inclusion criteria. We included only publications regarding PEComas (e.g., we did not include publications regarding angiomyolipoma etc.). The criteria for exclusion that were used precluded articles in the form of reviews, case-reports and those published in languages other than English. All included articles were assessed by two reviewers (GL and MP) for their compatibly for inclusion. In case of disagreement, a third reviewer (RM) was consulted.

## Results

### Available literature

There were 4278 Mesh retrieved publications for ‘PEComa’. A combination of this search with the Mesh ‘gynecologic neoplasm’ resulted in 162 publications. Of those, 137 had a Web of Science registry. Of those 132 were in English. After exclusion of non PEComa publications—there were 70 publications. Of those 58 case reports, seven reviews and five studies (Fig. 1).

A total of 84 patients were reported in 58 case reports. Most case report reported one patient (*n* = 46, 79.3%). The median age of patients reported in the case reports was 45 (range 11–80). Uterine PEComa were reported in 68 patients (81%), cPEComa of the cervix in eight (9%) patients, ovarian PEComa in five (6%) patients, vaginal PEComa in three (4%) patients. *Clinical highlights from case reports.*

A case report described uterine rupture in a 44 years old women, and another in a pregnant women 30 years old at 34 weeks of gestation [8, 9].

A report described an incidental PEComa found during cesarean delivery [10] in a term 38 year old women. A completion hysterectomy was performed during a second surgery and no residual disease was observed and the patient remained with no recurrence during the 7 months of follow-up.

Uterine sparing surgery was also reported [11] with removal of the mass as a myomectomy. This patient later underwent a cesarean delivery and did not recur during her follow up of 71 months.

A unique report described a PEComa in a 26 years old women that was found on the retained products of conception 10 days after a normal delivery [12].

A report of two cases concluded that MRI as unable to distinguish PEComas [13].

Time to metastasis was as short as 77 days from surgery [14] and varied up to 20 years for pulmonary nodules[15]. Recurrence from ovarian PEComa was reported at 25 months [16].

### Studies included

In a pathological study of malignant PEComa (*n* = 15), it was underlined that PEComacan be misdiagnosed as uterine sarcoma [17]. The median age was 56 years (range 27–86). mTOR alternations were present in 47%. Additional mutations were noticed in: TSC1 (27%) and TSC2 (20%). TP53 (53%), RB1 (30%), ATRX (33%), and BRCA2 (13%). Refarding treatment, nine patients received mTOR inhibitors. In terms of survival, 36% developed local recurrence and 71% (including the 36%) developed metastatic disease, most commonly to the lungs. Overall, 40% of these patients had metastatic disease at initial presentation. The median time for development of metastases was nine months. Two patients died of their disease at 16 and 38 months. That study doesn’t specify treatments such as chemotherapy, radiotherapy, and immune checkpoint inhibitors.

Another Study of 15 patients, reported a median age 61 years (range 43–71) [18]. Eight (53.3%) patients were diagnosed at stage I. Nine (60%) patients had recurrence (three in the peritoneum, tqo in lungs, two in soft tissue and on in the liver). Seven patients were treated with mTOR inhibitors, with best response being stable disease with LY3023414 (PI3K/mTOR inhibitor), and partial response with Temsirolimus. Six (40%) patients had no evidence of disease at the end of follow up, six (40%) had died of their disease, and three (20%) were alive with disease. The most common genetic mutations were as follows: TP53 (41% mutation, 12% deletion), TSC2 (29% mutation, 6% deletion), RB1 (18% deletion), ATRX (24% mutation), MED12 (12% mutation), BRCA2 (12% deletion), CDKN2A (6% deletion) as well as FGFR3, NTRK1 and ERBB3 amplification (each 6%).

In a study aiming to distinguish PEComas from uterine smooth muscle tumors, twenty-one uterine PEComas and 45 SMTs were analyzed for PNL2; HMB45, Melan-A, Cathepsin-K, Desmin, and h-Caldesmon [19]. Eighteen of 21 cases (86%) were positive for PNL2. All cases (21/21) were positive for HMB45. Over half of the PEComas (57%) were immunoreactive to Melan-A. The three PNL2 negative PEComas were all positive for HMB45, and 2/3 these cases were also Melan-A positive. All PEComas were positive for Cathepsin-K. Desmin was positive in 90% of PEComas and h-Caldesmon was positive in 57% of cases. That study concluded that PNL2 is a reliable biomarker for the diagnosis of uterine PEComa, with comparable sensitivity and specificity to HMB45, and greater sensitivity and extent of staining when compared with Melan-A.

A case series of thirty-two cases [20], dichotomized PEComas to two distinct patterns: classic appearance (majority of cases) and Lymphangioleiomyomatosis appearance. The median age of classic appearance was 51 years (range 32–77). Extrauterine disease was noted in 17% (5/30), with pulmonary metastases being most common. HMB-45 and cathepsin K were positive in all PEComas. Melan-A and MiTF were expressed in 77% and 79%, respectively. Each PEComa was positive for at least one muscle marker, with smooth muscle actin being the most common, followed by desmin and h-caldesmon. The median follow-up was 20 months with 63% of patients alive and well, 20% dead of disease, 13% alive with disease, and 3% dead from other causes. Recurrences occurred in 30%, with an average progression-free survival of 19 (range 2 to 65) months. On a univariate analysis, tumor size ≥ 5 cm, high-grade nuclear atypia, necrosis, mitoses > 1/50 HPFs, and lymphovascular invasion, were associated with aggressive behavior.

In a study of 16 cases [21] with a median age of 50 years(range 28–60), thirteen were originating from the uterus, two from the adnexa, and one from the vagina. In a mean follow up of 26 months, three patients died of disease, six were alive with disease, and seven were alive without evidence of disease at last follow-up. All patients who recurred or died had at least two of the following: size > 5 cm, high-grade nuclear features, infiltration, necrosis, lymphovascular invasion, or a mitotic rate > 1/50 high-power fields. HMB45 was universally expressed, followed by microphthalmia transcription factor (92%), MelanA (88%), and S100 protein (20%). Of the smooth muscle markers, desmin was universal (100%), followed by SMA (93%) and h-caldesmon (92%) and TFE3 (38%).

A subset of PEComas, the TFE3 translocation-associated PEComa [22], lack TSC mutation which may lead to hypothetical ineffectiveness of mTOR inhibitor therapy. The median age of the six patients in the series was 50 (range 46–66). Three cases arose in the uterus, one in the vagina, and one pelvic tumor and one pulmonary tumor, metastasis. Follow up ranged 1 to 57 months. Three cases demonstrated aggressive behavior and three cases had no evidence of recurrence.

Surgical resection of a uterine and vaginal PEComa, led to absence of disease recurrence at 9 and 5 months respectively, while a retroperitoneal PEComa recurred at 72 months in the retroperitoneum and lungs. Treatment with sirolimus was well tolerated and led to partial response [23].

### mTOR inhibitors and other treatments

The first report on the use of mTOR inhibitor temsirolimus in two metastatic patients, was in 2010 [24]. Since then, several case series reported on the use of MTOR inhibitors [25], including two out of three patients with advanced disease showing significant and prolonged response after extensive debulking surgery. One patient who progressed after response on temsirolimus—was switched to sirolimus, with a complete response and the patient remains disease-free. A case report described a remarkable complete response for the VEGFR inhibitor, Sorafenib, with the mTOR inhibitor, Sirolimus [26]. Table 1 summarizes main evidence for treatment with mTOR inhibitors.

**Table 1:** Main evidence for treatment with mTOR inhibitors

Source	Treatment	Number of patients (GYN/total)	Response rate	PFS	OS	Biomarkers for response
NCCN guidelines[5]	Albumin-bound sirolimus Sirolimus Everolimus Temsirolimus					
Wagner et al.[33]	nab-sirolimus 100 mg/m^2^ IV once weekly for 2 weeks In 3-week cycles	15/31	39%	10.6 months	40.8 months	TSC2-inactivating mutation pS6 expression
Bissler et al.[34] (Lymphangioleiomyomatosis)	Blood sirolimus level up to 10—15 ng per milliliter	-/20	100% response Reduction in size 46.3%—60.2% of initial mass size	N/A	N/A	N/A
Davies et al.[35] (Renal angiomyolipoma)	Blood sirolimus level up to 3—10 ng per milliliter	-/16	50%			
McCormack et al. [36] (Lymphangioleiomyomatosis)	Blood sirolimus level up to 5—15 ng per milliliter	-/46	Improvements in forced vital capacity: mean change of 230 ml			
Benson et al.[6]	Sirolimus 3 -5 mg orally. Temsirolimus weekly 25 mg IV	2/10	1 stable disease 1 partial response		Median 2.4 years	

Chemotherapy used for PEComa was ifosfamide, carboplatin and epirubicin [11, 27]. Some authors report also paclitaxel [28] and Imatinib following radiotherapy. Interestingly, there is a report of acute lymphoblasic leukemia following treatment by vincristine, ifosfamide, and anthracycline and radiotherapy comprising 45 Gy in an 11 years old with a uterine PEComa [29]. In a multicenter large report of PEComa patients treated with chemotherapy, out of 53 patients, 37 were female and of those—11 were with a uterine PEComas [30]. For Anthracycline-based chemotherapy (*n* = 23), the objective response rate was 13% with a median PFS of 3.2 months. For Gemcitabine-based chemotherapy (*n* = 15) the objective response rate was 20% with a median PFS of 3.4 months. Of note, in that study, the objective response rate for mTOR inhibitors was 41% with a PFS of 9 months. This underscores the low rate of response and the modest contribution for PFS for chemotherapy in gynecologic PEComa.

This review aimed to conglomerate the data available from the few studies regarding gynecologic PEComa, and to provide an accessible source to the data available, excluding case-reports and review papers which repeat published data, making access to the granular data more complicated. We do provide a source of reference to the known data from studies and the case series reported, with some important issues from published case-reports. However, this systematic-review has limitations.

This systematic review is not a meta-analysis and we did not evaluate for risk of bias. Another limitation in our review is the performance of the search on PubMed platform alone. This may exclude publications which are not indexed. However, it should be acknowledged that publication not indexed in PubMed may arise from journals with a different levels of peer-review process and of various qualities. Moreover, we did not register the protocol for this review before the conduction of this systematic review. While it is not mandatory to have a registered protocol, it is recommended by the Cochrane guidelines and this should be acknowledged.

## Discussion

Our systematic updated literature review has identified paucity of quality published work. Due to the rarity of gynecologic PEComas, multicentric studies would be needed to determine the role of newer therapeutic agents in PEComa. The creation of an international registry of PEComa and the standardization of the information provided in case series and case reports may improve their usefulness to produce valuable and helpful clinical and pathological data.

Molecularly, most PEComas, harbor a loss of function of the TSC1/TSC2 complex. loss of heterozygosityin the TSC2 gene, leads to activation of mTORC1 and disrupted cell growth signaling [31]. Finally, the endpoint of these mutations leads to activation of downstream pathways, such as the PI3K/AKT/mTOR pathway. There are possibly two primary molecular subtypes within PEComas. The initial subtype is characterized by uncontrolled activation of the mTORC1 pathway, whereas the second subgroup exhibits heightened transcriptional activity of TFE3, leading to the initiation of pro-oncogenic pathways such as c-Met, AKT, and mTOR [32].

## Conclusion

PEComas are rare and characterized by their distinctive histological features, leading to diagnostic and management challenges. While most gynecologic PEComas are benign, unique subsets exhibit aggressive behavior, emphasizing the importance of accurate diagnosis and individualized treatment strategies. Based on the mutational pattern, mTOR inhibitors have shown promising responses and further molecular understanding will allow us to evaluate other targeted treatments.

## Abbreviation

PEComa: Perivascular epithelioid cell neoplasms

## References

[1] Pea M (1996). Perivascular epithelioid cell. Am J Surg Pathol.

[2] Bonetti F (1992). PEC and sugar. Am J Surg Pathol.

[3] Bennett JA, Oliva E (2021). Perivascular epithelioid cell tumors (PEComa) of the gynecologic tract. Genes Chromos Cancer.

[4] Folpe AL (2005). Perivascular epithelioid cell neoplasms of soft tissue and gynecologic origin: a clinicopathologic study of 26 cases and review of the literature. Am J Surg Pathol.

[5] von Mehren M (2022). Soft tissue sarcoma, version 22022, nccn clinical practice guidelines in oncology. J Natl Compr Canc Netw.

[6] Liu Y (2021). Coexistence of conventional leiomyoma, fumarate hydratase-deficient atypical leiomyoma, and perivascular epithelioid cell tumor in a uterus: a case study. Int J Gynecol Pathol.

[7] Page MJ (2021). The PRISMA 2020 statement: an updated guideline for reporting systematic reviews. BMJ.

[8] Nguyen JMV (2020). Uterine rupture: an unusual presentation of a uterine perivascular epithelioid cell tumor (PEComa). Int J Gynecol Cancer.

[9] Nitahara K (2019). Rupture of perivascular epithelioid cell neoplasm at 34 weeks' gestation: a nonendometriosis case of spontaneous hemoperitoneum in pregnancy. J Obstet Gynaecol Res.

[10] Poomtavorn Y (2014). Caesarean section unmasking perivascular epithelioid cell tumour of the uterus. J Obstet Gynaecol.

[11] Shan W (2019). Five cases of uterine perivascular epithelioid cell tumors (PEComas) and review of literature. Arch Gynecol Obstet.

[12] Tilstra M (2015). Perivascular epithelioid cell tumor masquerading as retained placenta. J Ultrasound Med.

[13] Nishio N (2019). MR findings of uterine PEComa in patients with tuberous sclerosis: report of two cases. Abdom Radiol (NY).

[14] Issat, T., et al. 2012. Rare case of uterine PEC-oma (perivascular epithelioid cell tumor) recurrence. Case report and literature review. Ginekol Pol. 83 7 552 554. 22880484

[15] Ascione A (2022). Extremely late-onset pulmonary metastasis from uterine PEComa. Pathologica.

[16] Gadducci A (2021). Primary perivascular epithelioid cell tumor (pecoma) of the ovary: a case report and review of the literature. Anticancer Res.

[17] Anderson WJ (2023). A clinicopathologic and molecular characterization of uterine sarcomas classified as malignant PEComa. Am J Surg Pathol.

[18] Selenica P (2021). Genomic profiling aids classification of diagnostically challenging uterine mesenchymal tumors with myomelanocytic differentiation. Am J Surg Pathol.

[19] Valencia-Guerrero A (2020). PNL2: a useful adjunct biomarker to hmb45 in the diagnosis of uterine perivascular epithelioid cell tumor (PEComa). Int J Gynecol Pathol.

[20] Bennett JA (2018). Uterine PEComas: a morphologic, immunohistochemical, and molecular analysis of 32 tumors. Am J Surg Pathol.

[21] Schoolmeester JK (2014). Perivascular epithelioid cell neoplasm (PEComa) of the gynecologic tract: clinicopathologic and immunohistochemical characterization of 16 cases. Am J Surg Pathol.

[22] Schoolmeester JK (2015). TFE3 translocation-associated perivascular epithelioid cell neoplasm (PEComa) of the gynecologic tract: morphology, immunophenotype, differential diagnosis. Am J Surg Pathol.

[23] Nicolás I (2019). Perivascular epitheliod cell tumors: study of three gynecological cases. Med Clin (Barc).

[24] Italiano A (2010). Treatment with the mTOR inhibitor temsirolimus in patients with malignant PEComa. Ann Oncol.

[25] Starbuck KD (2016). Treatment of advanced malignant uterine perivascular epithelioid cell tumor with mTOR Inhibitors: single-institution experience and review of the literature. Anticancer Res.

[26] Gao F (2016). Combination targeted therapy of VEGFR inhibitor, sorafenib, with an mTOR inhibitor, sirolimus induced a remakable response of rapid progressive Uterine PEComa. Cancer Biol Ther.

[27] Cao B, Huang Y (2022). Malignant perivascular epithelioid cell tumor (PEComa) of the uterus. BMC Womens Health.

[28] Ciarallo A (2011). Malignant perivascular epithelioid cell tumor (PEComa) of the uterus: serial imaging with F-18 FDG PET/CT for surveillance of recurrence and evaluation of response to therapy. Clin Nucl Med.

[29] Jeon IS, Yi DY (2009). Acute lymphoblastic leukemia secondary to chemoradiotherapy for perivascular epithelioid cell tumor of uterus. Pediatr Hematol Oncol.

[30] Sanfilippo R (2019). Role of chemotherapy, VEGFR inhibitors, and mtor inhibitors in advanced perivascular epithelioid cell tumors (PEComas). Clin Cancer Res.

[31] Dickson MA (2013). Extrarenal perivascular epithelioid cell tumors (PEComas) respond to mTOR inhibition: clinical and molecular correlates. Int J Cancer.

[32] Utpatel K (2020). Complexity of PEComas : diagnostic approach, molecular background, clinical management. Pathologe.

[33] Wagner AJ (2021). Sirolimus for patients with malignant perivascular epithelioid cell tumors. J Clin Oncol.

[34] Bissler JJ (2008). Sirolimus for angiomyolipoma in tuberous sclerosis complex or lymphangioleiomyomatosis. N Engl J Med.

[35] Davies DM (2011). Sirolimus therapy for angiomyolipoma in tuberous sclerosis and sporadic lymphangioleiomyomatosis: a phase 2 trial. Clin Cancer Res.

[36] McCormack FX (2011). Efficacy and safety of sirolimus in lymphangioleiomyomatosis. N Engl J Med.

[37] Benson C (2014). A retrospective study of patients with malignant PEComa receiving treatment with sirolimus or temsirolimus: the Royal Marsden Hospital experience. Anticancer Res.

